# Supplementary material for: Next-Generation Influenza Vaccines and the Pandemic Horizon: Challenges, Innovations, and the Road Ahead
Source: Vaccines (Basel). 2025 Oct 27;13(11):1097. doi: 10.3390/vaccines13111097 (PMC12656365; doi:10.3390/vaccines13111097)
Supplement: Supplementary file 1 [file vaccines-13-01097-s001.zip › vaccines-3921461-supplementary.pdf]

Supplementary Materials

Supplementary S1. Online Survey Questions

The survey online was designed so that participants only needed to enter the name of their product(s) and its(their) vaccine platform(s) once. This information carried through the survey and allowed information on multiple products (for those developers providing information on more than one) to be captured.

1) Please indicate on which product(s) you are providing information in this survey by providing the name(s) of the next-generation influenza vaccine candidate(s).

2) Please indicate which platform(s) is being used for the vaccine(s) named above (Viral Vector, Recombinant protein, Influenza virus-based, Virus-like particles (VLP), Non-VLP nanoparticles, Nucleic acid-based – mRNA or saRNA, Nucleic acid-based – DNA, Other). If Other, please describe.

3) Please indicate if the product(s) named above are currently inactive/have discontinued product development. (yes or no)

4) Please indicate if the product(s) named above are in preclinical development. (yes or no)

Vaccine Platform Pros and Cons

5) What are favorable characteristics associated with using <*vaccine platform name*> as a vaccine platform with respect to next-generation influenza vaccine development (characteristics may be considered anticipated in some cases)? Please choose top five.

- Strong immunogenicity
- Elicits both humoral and cell mediated immune response
- Durable immunity (>1 year)
- Single-dose effectiveness
- Efficient route of administration (i.e., intramuscular, intranasal, etc.)
- Ease of production and scalability
- Adaptability for different antigens in the platform

- Vaccine safety profile/limited side effects
- Thermo-stability/ease of storage
- Rapid production
- Flexibility in formulation and valence
- Ability for timing of strain selection to occur closer to influenza season
- Targeted immune response/specificity
- Reduced risk of viral mutations/adaptations
- Rapid product development
- Cost-effectiveness
- Public acceptance
- Capacity for production to achieve vaccine equity
- Lower production costs
- Other: ____________

6) What are common challenges associated with using <*vaccine platform name*> as a vaccine platform with respect to influenza vaccine development (characteristics may be considered anticipated in some cases)? Please choose top five.

- Immune response interference against vector/pre-existing immunity to vaccine vector/platform
- Risk of adverse reactions
- Manufacturing challenges
- Cold chain/Storage requirements/stability
- Low immunogenicity, adjuvants and/or multiple does needed
- Cost of development
- Cost to manufacture
- Design complexity
- Limited antigenic coverage
- Complex production process
- Immune response limited to humoral vs cell-mediated
- Regulatory challenges
- Specialized equipment/platform required
- Immune response profile requires different measures of efficacy/immunogenicity than currently licensed influenza vaccines
- Public acceptance challenges/vaccine hesitancy
- Limited pre-existing data on safety and effectiveness
- Potential immune response variability in population, especially in young children or older adults
- Other: ________

Preclinical Development

7) Please identify the top three challenges and enablers associated with the **preclinical development** of the vaccine(s) named above, focusing on issues that challenge(d) or enable(d) movement through preclinical testing and to clinical development.

Challenges

a) ______________________________
b) ______________________________
c) ______________________________

Enablers

d) ______________________________
e) ______________________________
f) ______________________________

Clinical Development

8) What factors might assist **clinical development** of the vaccine(s) named above? Please choose top three.

Factors that might assist clinical development:

- Robust research infrastructure, including well-equipped laboratories, experienced researchers, and established clinical trial or human challenge study networks
- Technological advancements, such as improved vaccine production methods, adjuvant development, and antigen characterization
- Basic research about influenza viruses, including their genetic variations, transmission patterns, and immune response
- Collaborative partnerships between pharmaceutical companies, research institutions, and regulatory agencies
- Clear and efficient regulatory pathways with specific guidance on requirements for approval, including for new influenza vaccine platforms and technology
- Existence of a Target Product Profile

- Adequate, predictable, and sustained funding
- Other: ___________________________

9) What factors might hinder **clinical development** of the vaccine(s) named above? Please choose top three.

Factors that might hinder clinical development:

- Influenza virus variability
- Bottlenecks in the vaccine production timeline such as influenza strains identification, isolation, and propagation and vaccine production processes optimization
- Unfeasible effectiveness and safety targets
- Limited or poor sustainability of funding
- Stringent regulatory requirements or delays in the regulatory approval process
- Low vaccine uptake and demand that discourages manufacturers from investing in the clinical development of new influenza vaccines
- Low participation in clinical trials from vaccine hesitancy
- Poor availability of appropriate and standardized influenza viruses for human infection models
- Lack of manufacturers or Good Manufacturing Practices (GMP) materials for clinical trials
- Other: ___________________________

10) Please provide any additional comments regarding factors enabling or hindering clinical development of the vaccine(s) named above.

____

____

11) Aside from promising clinical trial results, what are the top 3 needs in order to advance the vaccine(s) named above to the next stage of **clinical development (**or product approval for those in phase 3 clinical trials)? Please fill in.

a) ____
b) ____
c) ____

12) If the vaccine(s) named above is trying to achieve multi-year protection, please describe how this will be demonstrated during clinical trials.

Partnerships

13) Have any product development partnerships or other agreements (e.g., technology transfer) been explored for the above listed influenza vaccine(s)? (yes or no)

If yes, please describe:

Regulatory

14) Have you engaged with a national regulatory agency to discuss the regulatory requirements for any of the vaccine candidate(s) named above? If you are providing information on more than one vaccine candidate, please answer yes if you have engaged with a national regulatory agency for any of the candidates. (yes or no)

If yes, please describe, indicating the vaccine candidate, which agency, and feedback received from that engagement (whatever information can be shared):

Clinical Development Time

15) Based on your current clinical development plans, please estimate when the vaccine(s) named above could be licensed.

Please indicate anticipated date for licensure (year) and/or time to licensure (number of years from current stage of development)

Influenza vaccine development and production leveraged for COVID-19

16) Did you leverage existing technical expertise, development efforts, and/or production capabilities from your influenza vaccine R&D program for COVID-19 vaccine development? (yes or no)

If yes, please describe:

____

____

Supplementary S2. In-depth Interview Questions

Global guidance on development of Next-Generation Influenza Vaccines

1) Have you consulted or used any guidance from funders or global health stakeholders (ex. WHO Preferred Product Characteristics, Gates Target Product Profile, United States National Institute of Allergy and Infectious Diseases Strategic Plan for a Universal Influenza Vaccine, Influenza Vaccine Research Roadmap) to inform the development of your product? If so, what did you use and why and how has it shaped development of your vaccine? If not, why not? Would other guidance materials be helpful?

Vaccine Platform Pros and Cons

2) On the survey, <*vaccine platform name*> platform was indicated for the next-generation influenza vaccine candidate. Why was this vaccine platform(s) chosen?

   You indicated that <(*refer to survey responses)*> were advantages and disadvantages of using this platform. Can you explain how this platform may impact development, production, and vaccine introduction? How can the challenges be addressed?

Preclinical Development

3) You indicated in the survey that <(*refer to survey responses)*> were the top three challenges and enablers associated with the preclinical development of <*name of next-generation influenza vaccine*>. Could you provide examples of how they have affected development of your vaccine?

Clinical Development

4) What challenges have you faced in clinical development of your product? Could you describe some examples of them?
5) Is <*name of next-generation influenza vaccine*> being tested in specific high risk populations? Can you explain more? When in the clinical development process would that happen?
6) Needle-free administration of vaccine products could be impactful in vaccination programs. Are delivery options in a needle-free form, for example microarray needle patches (MAPs), being considered?
7) How may introduction of next generation influenza vaccines impact production of currently available influenza vaccines?

Partnerships

8) Has your company/development group participated in product development partnerships or other agreements (ex. technology transfer) for current or next generation influenza vaccines?

Regulatory

9) How could regulatory guidance and clinical evaluation requirements for next-generation influenza vaccines, including for demonstrating multi-year protection, be improved? Has any guidance on how to evaluate <*name of vaccine*> been received? If so, could you share what it was?

Clinical Development Time and Cost Estimates

10) Could you estimate the cost of development thus far, indicating what stage of development you are at, and anticipated costs required to develop the vaccine further?
11) Is there an anticipated price point for <*name of vaccine*>? What are ways to increase accessibility for LMICs?

Pandemic Considerations

12) How could <*name of vaccine*> contribute to global pandemic preparedness and response efforts?
